# Automatic Sequence-Based Network for Lung Diseases Detection in Chest CT

**DOI:** 10.3389/fonc.2021.781798

**Published:** 2021-12-02

**Authors:** Jinkui Hao, Jianyang Xie, Ri Liu, Huaying Hao, Yuhui Ma, Kun Yan, Ruirui Liu, Yalin Zheng, Jianjun Zheng, Jiang Liu, Jingfeng Zhang, Yitian Zhao

**Affiliations:** ^1^ Cixi Institute of Biomedical Engineering, Ningbo Institute of Material Technology and Engineering, Chinese Academy of Sciences, Ningbo, China; ^2^ School of Optical Technology, University of Chinese Academy of Sciences, Beijing, China; ^3^ Hwa Mei Hospital, University of Chinese Academy of Sciences, Ningbo, China; ^4^ School of Medicine, Ningbo University, Ningbo, China; ^5^ Department of Eye and Vision Science, University of Liverpool, Liverpool, United Kingdom; ^6^ Department of Computer Science and Engineering, Southern University of Science and Technology, Shenzhen, China; ^7^ Zhejiang International Scientific and Technological Cooperative Base of Biomedical Materials and Technology, Ningbo Institute of Material Technology and Engineering, Chinese Academy of Sciences, Ningbo, China; ^8^ Zhejiang Engineering Research Center for Biomedical Materials, Ningbo Institute of Material Technology and Engineering, Chinese Academy of Sciences, Ningbo, China

**Keywords:** deep learning, CT, CNN, ConvLSTM, lung diseases

## Abstract

**Objective:**

To develop an accurate and rapid computed tomography (CT)-based interpretable AI system for the diagnosis of lung diseases.

**Background:**

Most existing AI systems only focus on viral pneumonia (e.g., COVID-19), specifically, ignoring other similar lung diseases: e.g., bacterial pneumonia (BP), which should also be detected during CT screening. In this paper, we propose a unified sequence-based pneumonia classification network, called SLP-Net, which utilizes consecutiveness information for the differential diagnosis of viral pneumonia (VP), BP, and normal control cases from chest CT volumes.

**Methods:**

Considering consecutive images of a CT volume as a time sequence input, compared with previous 2D slice-based or 3D volume-based methods, our SLP-Net can effectively use the spatial information and does not need a large amount of training data to avoid overfitting. Specifically, sequential convolutional neural networks (CNNs) with multi-scale receptive fields are first utilized to extract a set of higher-level representations, which are then fed into a convolutional long short-term memory (ConvLSTM) module to construct axial dimensional feature maps. A novel adaptive-weighted cross-entropy loss (ACE) is introduced to optimize the output of the SLP-Net with a view to ensuring that as many valid features from the previous images as possible are encoded into the later CT image. In addition, we employ sequence attention maps for auxiliary classification to enhance the confidence level of the results and produce a case-level prediction.

**Results:**

For evaluation, we constructed a dataset of 258 chest CT volumes with 153 VP, 42 BP, and 63 normal control cases, for a total of 43,421 slices. We implemented a comprehensive comparison between our SLP-Net and several state-of-the-art methods across the dataset. Our proposed method obtained significant performance without a large amount of data, outperformed other slice-based and volume-based approaches. The superior evaluation performance achieved in the classification experiments demonstrated the ability of our model in the differential diagnosis of VP, BP and normal cases.

## 1 Introduction

COVID-19, the latest in viral pneumonia diseases, is an acute respiratory syndrome that has spread rapidly around the world since the end of 2019, having a devastating effect on the health and well-being of the global population (1, 2). To diagnose viral pneumonia (limited to COVID-19 in our work), reverse transcription-polymerase chain reaction (RT-PCR) has widely been accepted as the gold standard. However, shortages of equipment and strict requirements for testing environments limit the rapid and accurate screening of suspected subjects. Furthermore, RT-PCR testing is also reported to suffer from a high false-negative rate (3), with a low sensitivity of only 71%. In clinical practice, radiological imaging techniques, e.g., X-rays and computed tomography (CT), have also been demonstrated to be effective in diagnosis, and also follow-up assessment and evaluation of disease evolution (4, 5). CT is the most widely used imaging technique, due to its high resolution and three-dimensional (3D) view, and its relatively high detection sensitivity of around 98% (6). For example, the study (5) found that the dynamic lesion process of viral pneumonia (from ground-glass opacity in the early stage to pulmonary consolidation in the late stage) can be observed in CT scans, and its CT manifestations have been emphasized.

**Figure 1:**
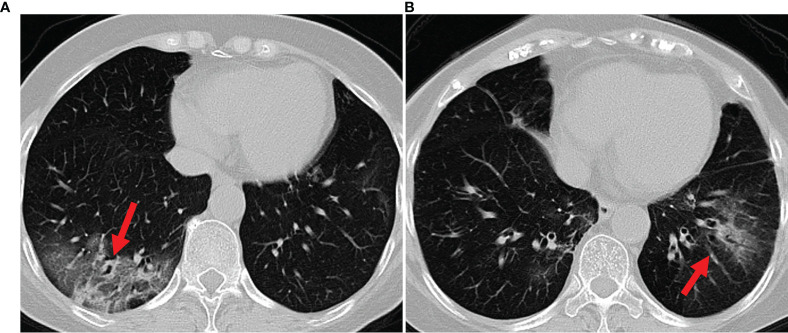
Example axial CT slices of viral pneumonia **(A)**, bacterial pneumonia **(B)**. Accurate classification of different types of pneumonia is imperative for timely diagnosis and treatment. However, viral pneumonia and bacterial pneumonia display similar appearances in a CT image, which makes it difficult to accurately differentiate a patient with viral pneumonia from a case of bacterial pneumonia.

Bacterial and viral pathogens are the two leading causes of pneumonia, but require very different forms of management (7). Bacterial pneumonia requires urgent referral for immediate antibiotic treatment, while viral pneumonia is treated with supportive care. Therefore, accurate classification of different types of pneumonia is imperative for timely diagnosis and treatment. However, the imaging features of viral and bacterial infections are not often compared, and the only imaging feature that was significantly different between the viral and bacterial lung infection was the frequency of diffuse airspace disease (8). In the case of a typical viral pneumonia, in clinical practice, it is difficult to accurately differentiate viral pneumonia from bacterial pneumonia. See **Figure 1** as an example. In clinical practice, especially in primary medical institutions, the consistency of imaging diagnosis of pneumonia pathogens is poor (9–11). Moreover, it is time consuming for radiologists to read CT volumes that contain hundreds of 2D slices. As such, it is of great practical significance to quickly and accurately identify pathogens to guide individualized anti-infectious treatment and minimize and delay the occurrence of drug resistance.

As an emerging technology in medical image analysis, artificial intelligence (AI) has been widely employed for lesion segmentation, and for clinical assessment and diagnosis of lung-related diseases *via* radiological imaging (12, 13). Recently, many novel AI techniques for viral pneumonia have been presented (1). For instance, Ouyang et al. (14) proposed a dual-sampling attention network for the differential diagnosis of COVID-19 from Community Acquired Pneumonia (CAP) (14, 15), and Fan et al. (16) introduced an automatic COVID-19 lung infection lesion segmentation method using a deep network. These works are useful for detecting and controlling of the spread of COVID-19. However, there are very few studies on differentiating COVID-19 from other etiological pneumonias, despite success in using deep learning (DL) approaches to discriminate bacterial and viral pneumonias in pediatric chest radiographs (17, 18).^^1^^

Further, most existing works make use of 2D CT slices, and the lack of continuity information makes it impossible to capture the true spatial distribution of the lesion in the lungs. To this end, some recent studies have attempted to use entire 3D volumes to train a 3D classification or segmentation model directly (14, 19) ,achieving a slightly better performance than the approaches based on 2D slices. However, these 3D volume based approaches greatly increase the computational load, and require much more powerful and expensive hardware configurations. Additionally, 3D volumes may contain large portions of redundant information, which leads to great difficulty in accurately identifying small lesions. The imaging appearance of viral and bacterial lung infection has considerable similarity, and that, in any individual case, the viral pneumonia cannot reliably be distinguished from bacterial infections (8, 20). For example, viral pneumonia and bacterial pneumonia have some image features in common, such as ground glass opacities and interstitial changes in the peripheral zone of lungs, and accompanied by partial consolidation. The only imaging feature that was significantly different between the viral and bacterial lung infection was the frequency of diffuse airspace disease (8). Precise characterization of the spatial morphology of the infected regional lesions is essential to distinguish the two infection types by CT imaging.

In this paper, we treat the spatially continuous 2D CT slices as a time sequence and proposed a unified sequence-based pneumonia classification network (SLP-Net) for differentiating viral pneumonia (VP) from bacterial pneumonia (BP) and normal control cases. Our network comprises a CNN encoder and the ConvLSTM module, and sequence attention maps are used for auxiliary classification. As stated above, the precise characterization of the lesion is the key to distinguish the different pneumonia types. The combination of these components ensures the model pay more attention to spatial morphology of the lesion during the decision making. Specifically, the encoder with multi-scale receptive fields is first used to extract local representations of the sequence. Then we apply the ConvLSTM to acquire spatial information of these sequence features, modeling the distribution of the lesion. To optimize the SLP-Net, we introduce a novel adaptive-weighted cross-entropy (ACE) loss, with a view to ensuring that as many valid features from the previous images as possible are encoded into the subsequent CT image. Given the fact that the final diagnosis conclusion needs to be made for each patient, case-based prediction rather than a slice- or sequence-based prediction is more valuable. To obtain case-based prediction, in addition to the classification result of the sequence, we also use sequence attention maps to aid the case-level classification, aiming to enhance the confidence of the results. We collect a dataset of 258 chest CT volumes (153 VP, 42 BP, and 63 normal control cases). The experimental results show that the proposed SLP-Net achieves an accurate classification performance of viral pneumonia, bacterial pneumonia, and normal control, which could benefit the large-scale screening and control of viral pneumonia, and also enable efficient treatment for different types of pneumonia.

We organize the remainder of this paper as follows. In Section 2, the existing methods of AI-enpowered viral pneumonia analysis are briefly reviewed. In Section 3 we give detailed descriptions of collected datasets. Section 4 introduces the proposed SLP-Net. In Section 5, we present the experimental results and discuss the effectiveness, robustness, and efficiency of the SLP-Net. Section 6 concludes the paper and indicates directions for future work.

## 2 Related Work

AI-based medical image analysis plays an essential role in the global fight against COVID-19, and a considerable number of approaches have been proposed in the past five months. This body of work on COVID-19 has focused primarily on two problems: lesion segmentation (16, 21, 22), and automated screening (23–31). For example (16), recently introduced a parallel partial decoder to aggregate high-level features, using an implicit reverse attention and explicit edge-attention to model boundaries and enhance representations so as to identify infected regions from 2D chest CT slices. To alleviate the shortage of labeled data, a semi-supervised segmentation framework based on a randomly selected propagation strategy was applied by (21). They proposed a relational approach, in which a non-local neural network module was introduced to efficiently learn both visual and geometric relationships among all convolutional features.

However, automated viral pneumonia (e.g., COVID-19) screening has attracted even more attention. For instance (32), introduced a COVID-19 detection method with multi-task DL approaches, using an inception residual recurrent convolutional neural network (CNN) with transfer learning. Their detection model achieved 84.67% accuracy from X-ray images (33). proposed a deep features fusion and ranking technique to detect COVID-19 in its early phase. They employed a pre-trained CNN structure to obtain a set of features, which were subsequently fused and evaluated with a support vector machine (SVM) classifier. In the classification task of COVID-19 and no COVID-19, their proposed method obtained 98.27% accuracy on their own dataset (34). applied a modified residual network, called DeepPneumonia, based on ResNet50 for slice-level classification, and could discriminate the COVID-19 patients from the bacteria pneumonia patients with an AUC of 0.95 (23). built multiple deep convolutional neural models for classifying chest X-ray images into normal and COVID-19 cases, which obtained 96.1% accuracy (35). proposed a unified latent representation to explore multiple features describing CT images from different views, a method that can completely encode information from different features aspects and is endowed with a promising class structure for separability. Performance in diagnosis for COVID-19 and community-acquired pneumonia (CAP) is 95.5% in terms of accuarcy. An infection size-aware random forest method was introduced by (15) for the differentiation of COVID-19 from CAP, in which patients were automatically categorized into groups with different extensions of infected lesion sizes, followed by generation of random forests with each group for classification. The method achieved an accuracy of 89.4% in discriminating COVID-19 from CAP.

However, all of the above mentioned works are based on 2D images, the spatial correlation between consecutive CT scans is neglected by most slice-based methods, despite this being essential for the screening of lung diseases. A variety of volume-based methods have been proposed in an attempt to address this deficiency (19). proposed an attention-based deep 3D multi-instance learning method to screen COVID-19 from 3D chest CT sacns, using a weakly supervised learning framework that incorporates an attention mechanism into deep multi-instance learning, achieving an accuracy of 97.9% (14). proposed a 3D CNN to diagnose COVID-19 from CAP, in which a novel online attention module is combined with a dual-sampling strategy. The online attention module focuses on the infected regions when making diagnostic decisions. The dual-sampling strategy mitigates the imbalanced distribution in the sizes of infected regions between COVID-19 and CAP. Their method was evaluated in private dataset and achieved an accuracy of 87.5%. These 3D volume based approaches greatly increase the computational load, and require much more powerful and expensive hardware configurations. Additionally, 3D volumes may contain large portions of redundant information, which leads to great difficulty in accurately identifying small lesions. Overall, 2D slice based methods cannot take advantage of spatial continuity information and 3D volume based methods require much more expensive hardware configurations. In order to take advantage of the complementary information of 2D slices and 3D volumes, we treat the spatially continuous 2D CT slices as a time sequence, and divide the volume into multiple different temporal sequences of consecutive slices as the input.

## 3 Materials and Methods

### 3.1 Materials

A total of 258 subjects were enrolled into this study, with 258 CT volumes, corresponding to 43,421 slices. Of the 258 subjects, 42 patients were confirmed positive for BP by clinical diagnosis (age: 59.5 ± 27.2; male/female: 36/6), 153 patients were positive for VP, confirmed by RT-PCR (age: 52.3 ± 12.7; male/female: 68/85), and 63 were control subjects (age: 35.8 ± 11.7; male/female: 33/30). The CT volumes of normal and VP patients were captured between January 29, 2020 and February 18, 2020, and the BP data was collected between January 2, 2019 and February 19, 2020. There is no statistically significant difference between the ages of the VP and BP subjects (*P* = >0.05), but both groups are significantly older than patients in the normal group (*P* < 0.001). CT examinations of all the enrolled patients were performed on a ScintCare CT16 (Minfound Inc, China) with standard chest imaging protocols. All the patients underwent CT scans during the end-inspiration without the administration of contrast material. Related parameters for chest CT scanning were listed as follows: field of view (FOV), 360 mm; tube voltage, 120 kV; tube current, 240 mA; helical mode; slice thickness, 5 mm; pitch, 1.5; collimation 16 × 1.2 mm; gantry rotation speed, 0.5 s/r; matrix, 512 × 512; software version, syngo CT 2014A; mediastinal window: window width of 350 HU, with a window level of 40 HU; and lung window: window width of 1,300 HU, with a window level of −500 HU.

CT volumes were retrospectively collected according to the history of laboratory investigations (e.g., sputum culture and reverse transcription-polymerase chain reaction), which we can generate the case-level labels. Meanwhile, professional radiologists (from the Hwa Mei Hospital, University of Chinese Academy of Sciences, Ningbo, China.) picked out the slice containing the infected region in each volume for the subsequent automatic generation of sequence labels: each volume was be divided into overlapping sequences containing *n* slices, with *k* overlapping slices between two sequences. If a sequence from VP volume contains the infected slice(s), the label for that sequence is 1; if it is from BP volume and contains infected slice(s), the label is 2; the label from normal volume is 0. It is worth noting that *k* and *n* are both hyperparameters, and in *Sensitivities to Hyperparameters* we discuss how to choose the values of these, as well as their impact on the classification results.

The sequences generated from the volume of VP and BP were not used for training if they did not contain any slices with lesion regions. If all normal slices from patients are used for training instead of excluding them, it will increase the proportion of normal control samples in the training set.The data imbalance may cause the model to over-fit the normal samples and tend to predict the samples with the lesion as normal. To avoid this, we exclude the normal slice from patients. For training and evaluation of the proposed method, as shown in **Table 1**, we split 258 volumes into 168 volumes (95 VP, 30 BP, and 43 normal controls) for training and 90 (58 VP, 12 BP, and 20 normal controls) volumes for testing.

**Table 1:** Characteristics of training and testing CT dataset for identifying viral pneumonia (VP) from bacterial pneumonia (BP) and normal controls.

Cohort	VP		BP		Normal controls		Total	
	Volumes	Slices	Volumes	Slices	Volumes	Slices	Volumes	Slices
Training set	95	15,931	30	5,068	43	7,310	168	28,309
Testing set	58	7,832	12	3,107	20	4,173	90	15,112
Total	153	23,763	42	8,175	63	11,483	258	43,421

### 3.2 Proposed Method

**Figure 2:**
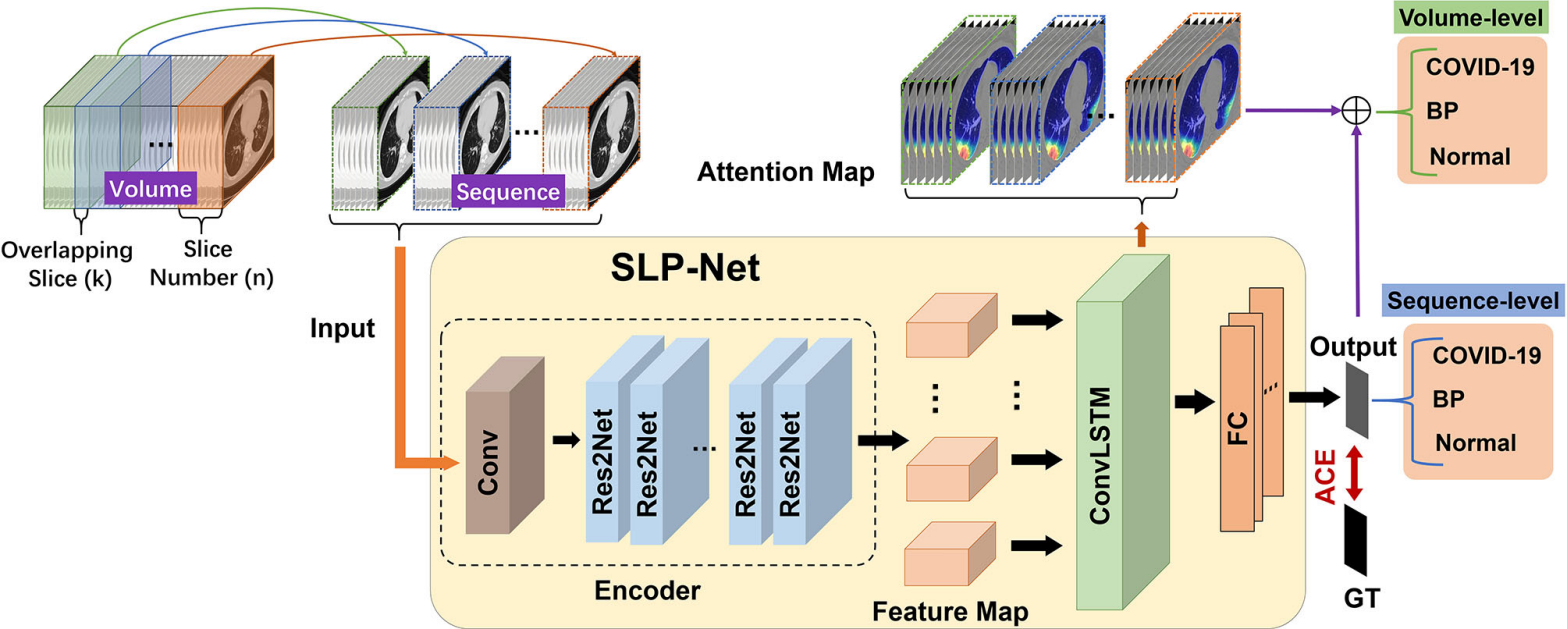
Flowchart of our whole system for differentiating between viral pneumonia and bacterial pneumonia in a chest CT volume. Each volume is divided into overlapping sequences containing *n* slices during the training phase, such that the overlapping slices between two sequences are *k*. When predicting each volume during the testing phase, in addition to using the model to obtain the classification results of the sequence, we also introduce sequence attention maps for auxiliary classification to enhance the confidence level of the results. GT, ground truth; ACE, adaptive-weighted cross-entropy loss; FC, fully connected layer.

The architecture of our SLP-Net is shown in **Figure 2**, which consists of two main components: sequence CNNs, and ConvLSTM. The sequence CNNs with multi-scale receptive fields are employed to extract more discriminative high-level features from the CT sequence, while the ConvLSTM captures the axial dimensional dynamics of features. In addition, a sequence attention map is utilized as an auxiliary means to integrate the output of the network and obtain prediction results with a higher level of confidence. Finally, an adaptive-weighted cross-entropy (ACE) loss is used to optimize the whole model.

#### 3.2.1 Sequence CNN With Multi-Scale Receptive Fields

A typical CNN model consists of a stack of convolution layers, interleaved with non-linear downsampling operations (e.g., max pooling) and point-wise nonlinearities (e.g., ReLU). The residual shortcut used in ResNet can reduce the over-fitting of the model, so that the depth of the network can be greater and achieve better performance. Taking into consideration the problems of overfitting and parameter cost, we employed ResNet (36) as our encoder backbone. The first four feature-extracting blocks are retained, without the average-pooling layer and the fully-connected layers.

**Figure 3:**
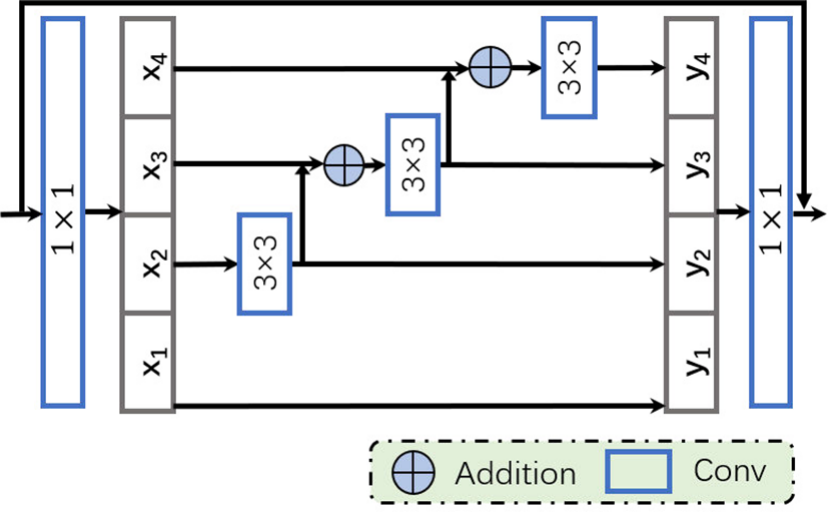
A Res2Net module is utilized to extract more discriminative features.

Since VP and BP reveal similar appearances in CT images, we aim to obtain more discriminative features by employing multi-scale information, in order to distinguish them more accurately. Unlike most existing methods (37–39) that improve multi-scale ability by utilizing features with different resolutions, we apply a recently proposed multi-scale receptive fields technique (40) to enhance representation ability at a more granular level. Specifically, we apply a modified bottleneck with multi-scale ability, the Res2Net module, to replace a group of 3 × 3 filters used in the original bottleneck block of ResNet. As shown in **Figure 3**, after the 1 × 1 convolution, feature maps are split into *s* feature map subsets, denoted by *x_i_*. Then, apart from *x*_1_, each *x_i_* goes through a corresponding 3 × 3 convolutional operator, denoted by *K_i_* (·), where *y_i_* is the output of *K_i_* (·). The output of *K_i–1_* (·)is added to *x_i_*, then sent to the next group of filters *K_i_* (·). Thus, *y_i_* can be defined as follows:


(1) $$y_{i}=\{\begin{array}{cc} x_{i} & i=1;\\ K_{i}(x_{i}) & i=2;\\ K_{i}(x_{i}+y_{i-1}) & 2<i\leq s. \end{array}$$

In order to better integrate the information from different scales, all outputs *y_i_*, where *i* ∈{1,2, …, *s*}, are concatenated and passed through a 1×1 convolution. Such splitting and concatenation strategies can force the convolution to process features more efficiently. Note that each 3×3 convolution operation *K_i_* (·)receives information from all the feature splits {*x_j_*, *j* ≤ *i*}. Each time *x_j_* performs a 3×3 convolution, the size of the receptive field will increase. Due to the combinatorial effect, the output of the Res2Net block contains different combinations of receptive field sizes/scales.

#### 3.2.2 ConvLSTM With ACE Loss

Although the conventional fully-connected LSTM (FC-LSTM) can handle sequences of any length and capture long-term dependencies (41), it contains too much redundancy for spatial data, which is a critical problem for image sequences. Inspired by video object detection (42), we apply ConvLSTM (43) to process the feature sequences from the encoder.

**Figure 4:**
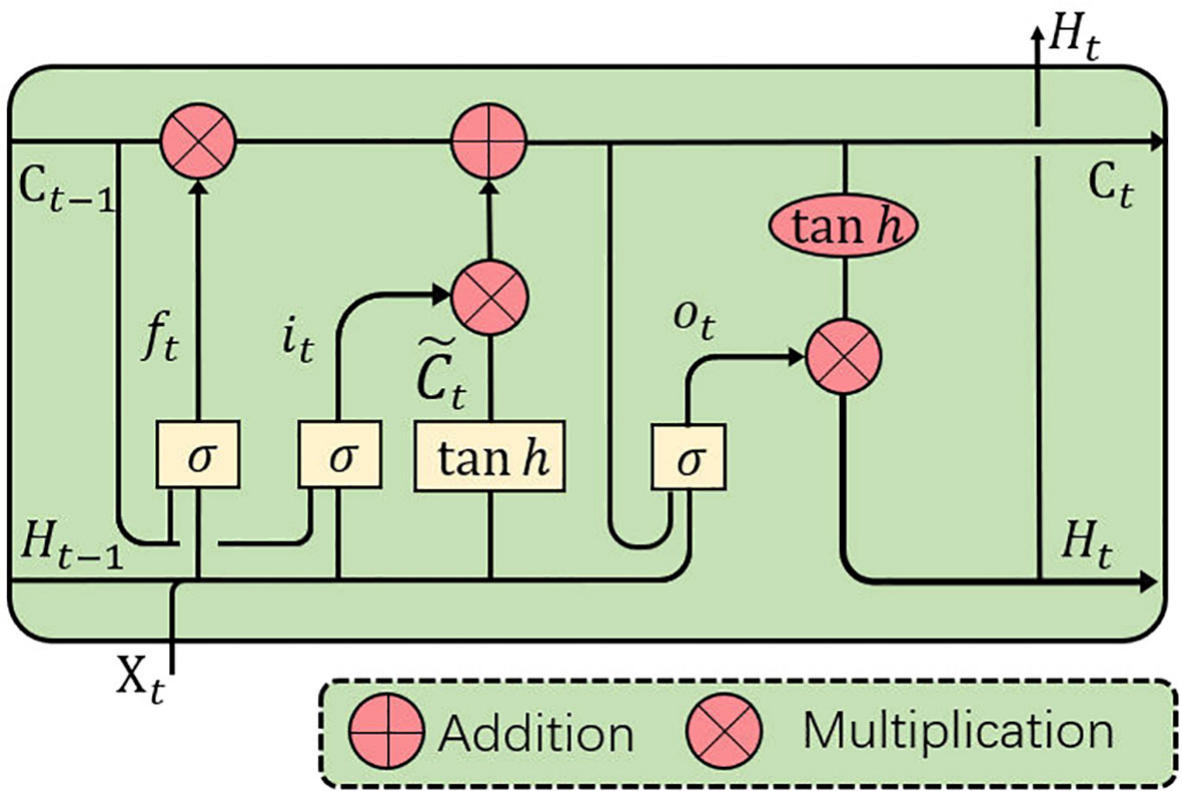
ConvLSTM is utilized to implicitly learn axial dimensional dynamics and efficiently fuse axial dimensional features.

As the convolutional counterpart of the FC-LSTM, the ConvLSTM introduces the convolution operation into the input-to-state and state-to-state transitions. The ConvLSTM can model axial dimensional dependencies while preserving spatial information. As with the FC-LSTM, the ConvLSTM unit (see **Figure 4**) includes an input gate *i_t_*, a memory cell *C_t_*, a forget gate *f_t_* and an output gate *o_t_*. The memory cell *C_t_*, acting as an accumulator of the state information, is accessed, updated and cleared through self-parameterized controlling gates: *i_t_*, *o_t_*, and *f_t_*. If the input gate is switched on, the new data is accumulated into the memory cell once an input arrives. Similarly, the past cell status *C_t–_*_1_ will be forgotten if the forget gate *f_t_* is activated. The output gate *o_t_* further controls whether the latest memory cell’s value *C_t_* will be transmitted to the final state *H_t_*. With the above definitions, the ConvLSTM can be formulated as follows:


(2) $$\begin{array}{c} i_{t}=\sigma (W_{xi}*X_{t}+W_{hi}*H_{t-1}+W_{ci}\;C_{t-1}+b_{i}),\\ f_{t}=\sigma (W_{xf}*X_{t}+W_{hf}*H_{t-1}+W_{cf}\;C_{t-1}+b_{f}),\\ C_{t}=f_{t}\;C_{t-1}+i_{t}\;\tanh(W_{xc}*X_{t}+W_{hc}*H_{t-1}+b_{c})\\ o_{t}=\sigma (W_{xo}*X_{t}+W_{ho}*H_{t-1}+W_{co}\;C_{t}+b_{o}),\\ {ℋ}_{t}=o_{t}\;\tanh(C_{t}), \end{array},$$

where ‘*’ denotes the convolution operator, ‘°’ denotes the Hadamard product, and *σ* is the sigmoid activation function. *X_t_* and *ℋ_t_* are the input and output of the ConvLSTM at time step *t* (*t* indicates the *t*th frame in a CT image sequence, and slices will be referred to as frames in the sequel.), and *i_t_*, *f_t_*, and *o_t_* indicate the input, forget and output gates, respectively. *b_i_*, *b_f_*, and *b_o_* are the bias of the input gate, forget gate, and output gate. A memory cell *C_t_* stores the historical information. All the gates *i*, *f*, *o*, memory cell *C*, hidden state ℋ and the learnable weights *W* are 3D tensors. Input sequences 𝒳 are fed into a ConvLSTM block, which captures the long and short-term memory of sequences and contains both axial dimensional information, for use in implicitly learning axial dimensional dynamics and efficiently fusing axial dimensional features.

We define *L_t_* as the output of the ConvLSTM layer at time step *t*. The output of the ConvLSTM layer is fed to the fully-connected (FC) layers, which transform the features into a space that makes the output easier to classify. The outputs of the FC layers are defined as *O_t_* at time step *t*. Ideally, the longer the image sequence, and the more classification information ConvLSTM processes, the higher the confidence of classification. From this perspective, it is sufficient to use the output of the final time step for classification without further processing. However, in practice, due to differences in the distribution of lesions on different slices, there may be some useful information that has not been accumulated in the memory cell. In order to enchance the memory ablity of ConvLSTM for CT sequence at different slices and ensure that as much valid information from the previous slices as possible are encoded, we propose to use all the intermediate outputs of every time step as our feature for identification. A better ConvLSTM means that the longer the sequence it processes and the more comprehensive information it considers, the more confident it identifies the input. From this perspective, instead of minimizing the loss on the final time step, we define a new adaptive-weighted cross-entropy (ACE) loss to use all the intermediate outputs of every time step weighted by *w_t_*:


(3) $${ℒ}_{ACE}=\frac{1}{n}\sum_{t=1}^{n}\sum_{p=1}^{P}-w_{t}[y_{p}\log(C_{p}(O_{t}))],$$

where *C* and *p* denote the classifier and classification label, respectively, and *n* denotes the number of images in a sequence. *C_p_* (*O_t_*) indicates the classifier *C*, which correctly identifies the final output *O* at time step *t*, *y_p_* ∈{0, 1, 2} are the label values; and *P*=3 denotes the total number of labels. Finally, *w_t_* is the weight of each frame in a sequence. weight. We let each group of two weight items constitute the arithmetic sequence.

Since the importance of the information contained in different slices is different, it is not reasonable to use the equal weights. Due to the output of the final time step has taken into account all other previous slices , it contains the most information, and the further away from the last output, the less information it contains. Moreover, the number of slices in a sequence is a hyper-parameter, we adopt an adaptive weighting scheme. The output of the final time step should be assigned the maximum weight, and the farther away from the final time step, the smaller the weight. Specifically, we let each group of two weight items constitute the arithmetic sequence: the sum of which is 1. The first two items are taken as 0.01, namely, *w*_1_ = *w*_2_ = 0.01, and the subsequent weights can then be calculated according to the hyperparameter *n* and weight *w*_1_. The ACE loss ensures that the features of the previous CT images in the sequence can be encoded into the later image.

#### 3.2.3 Auxiliary Diagnosis With Attention Maps

**Figure 5:**
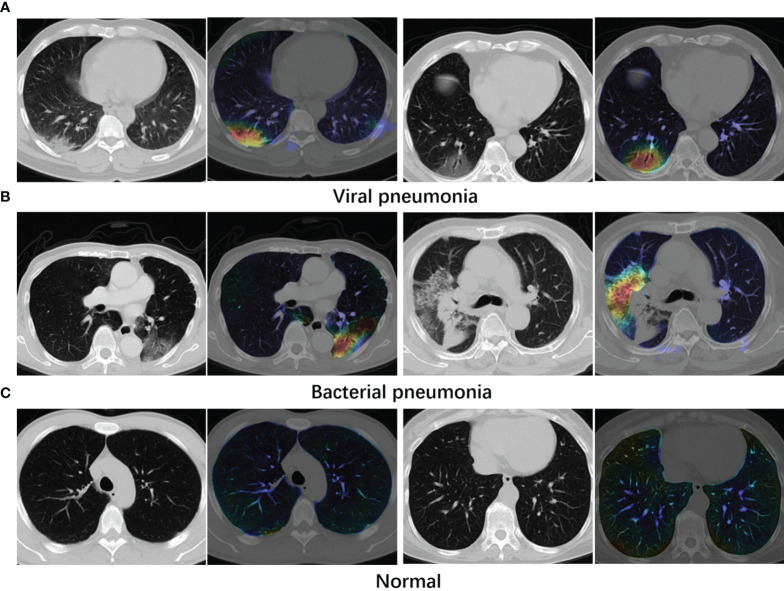
Examples of attention maps obtained with Grad-CAM. **(A)** Viral pneumonia cases. **(B)** Bacterial pneumonia cases. **(C)** Normal cases. Lighter colors indicate the stronger response regions. From the maps, the infected regions receive greater attention.

Deciding which type (VP, BP, or normal) the entire volume belongs to based on the prediction results of the sequence is a critical step in auxiliary diagnosis. For higher confidence, in addition to using the model to obtain the classification result of the sequence, we also utilized the Grad-CAM (44) technology to generate attention maps of the sequence to assist the prediction. Grad-CAM is a method for producing visual interpretations for CNNs in the form of class-specific saliency maps. A saliency map, $L_{t}^{c}$ , is produced for each image input based on the activation from *k* filters, $A_{ij}^{k}$ , at the final convolutional layer. To make the method applicable to image sequences, the activations for all timesteps *t* in the sequence are considered (Eq. R1).


(R1) $$L_{ijt}^{c}=\sum_{a}w_{kt}^{c}A_{ijt}^{k};\;w_{kt}=\frac{1}{Z}\sum_{ij}\frac{\partial F^{c}}{\partial A_{ijt}^{k}}$$

where *Z* is a normalizing constant and *F^c^* is the network output for the class *c*. *i, j* are pixel location of filter *A^k^*. In the visualization examples shown in **Figure 5**, stronger class activation map (CAM) areas are indicated with lighter colors.

During the prediction phase, we can obtain the classification results of sequences belonging to a volume. In addition, we apply Grad-CAM to generate the response heat map of each sequence. A volume containing *m* sequences will be classified as viral pneumonia (VP) sample if it meets both of following criteria: (a) More sequences are classified as viral pneumonia (VP) then bacterial pneumonia (BP) in this volume; (b) There are two adjacent sequences with the category of viral pneumonia whose activation regions have an intersecting area of more than 50%. If the second criterion is not satisfied for VP, the bacterial type sequences are checked if there are two adjacent sequences with an intersecting area of more than 50% of the activation region, and if so the sample is classified as bacterial type, otherwise it is classified as normal. Notably, if there are the same number of VP and BP sequences, the one with the greater average sequence probability value is treated as the dominant category. The possibility output of the network dominates the classification on the volume level, and Attention Map is used as an auxiliary during the decision making.

### 3.3 Evaluation Metrics

We employ the commonly used metrics for multi-class classification to measure performance: e.g., weighted sensitivity (Sen, also known as recall), specificity (Spe), accuracy (Acc), and balanced accuracy (B-Acc, a.k.a. balanced classification rate). In order to reflect the tradeoff between sensitivity and specificity, and evaluate the quality of our classification results more reliably, a kappa analysis and F-measure (*F*1 score) are also provided following (45). These two measures are more robust than other percentage agreement measures, as they take into account the possibility of the agreement occurring by chance. The weighted sensitivity (Sen), specificity (Spe), accuracy (Acc), and balanced accuracy (B-Acc) are defined as:


$$Spe=\sum_{i=1}^{P}w_{i}\frac{TN_{i}}{TN_{i}+FP_{i}},\;Sen=\sum_{i=1}^{P}w_{i}\frac{TP_{i}}{TP_{i}+FN_{i}},$$

$$B-Acc=\frac{\left(Sen+Spe\right)}{2},\;Acc=\sum_{i=1}^{P}w_{i}\frac{TP_{i}+TN_{i}}{TP_{i}+FN_{i}+FP_{i}+TN_{i}},$$

where TP*_i_* indicates the number of true positives, TN*_i_*—the number of true negatives, FP*_i_*—the number of false positives, and FN*_i_*—the number of false negatives for the i–*th* classification label; and w*_i_* represents the percentage of images whose ground truth labels are *i*. The kappa values and F-measure (*F*1 score, a.k.a. Dice score) are defined as follows:


$$p_{o}=\sum_{i=1}^{P}w_{i}\frac{TP_{i}}{n},\;p_{e}=\sum_{i=1}^{P}\frac{a_{i}*b_{i}}{n*n},\;Pre=\sum_{i=1}^{P}w_{i}\frac{TP_{i}}{TP_{i}+FP_{i}},$$

$$Kappa=\frac{p_{o}-p_{e}}{1-p_{e}},\;F1=2\cdot \frac{Pre\cdot Sen}{Pre+Sen},$$

where a*_i_* denotes the true sample number of each class, b*_i_* denotes the predicted sample number of each class, *n* denotes the total sample number, and *P* denotes the number of classes. Note that *kappa* values between 0.81 to 1.00 indicate almost perfect agreement, values between 0.61 and 0.80 exhibit substantial agreement, values of 0.41–0.60 exhibit moderate agreement and values less than 0.40 exhibit poor to fair agreement. The *F*1 score reaches its best value at 1 and worst at 0. We also present the ROC curves and the area under ROC curve (AUC) for VP against BP.

### 3.4 Implementation Details

The proposed method was implemented in the publicly available Pytorch library. The combination of CNN and ConvLSTM makes the model more complex. To accelerate convergence, we first trained a CNN classification network with a labeled 2D slice. After removing the FC layer, the encoder is used as the initialization parameter of SLP-Net. During the training phase of SLP-Net, CNN and ConvLSTM are jointly trained in an end-to-end manner using Adam optimizer. In practice, we found that CNN pre-training does speed up the convergence of the model, but has no effect on the final classification performance. The learning rate was gradually decreasing starting from 0.0001, and the momentum was set to 0.9. In addition, online data enhancement was employed to enlarge the training sequence data. The same data enhancement was used for all images in a sequence: we implemented data augmentation in a random way, including brightness, color, contrast, and sharpness transformation from 90 to 110%. We set a random seed from 1 to 4 for the enhancement.

## 4 Results

### 4.1 Classification Performances

To compare the classification performance, we evaluated the detection ability of the model at both sequence and volume levels. All the existing pneumonia detection methods are accomplished using 2D CT slices or 3D volumes. To further verify whether the features containing both axial dimensional and spatial information captured by our model could benefit detection performance, we compared the proposed method to other classic classification models using 2D slices: AlexNet (46), VGG19 (47), InceptionV3 (48), ResNet34 (36), and Xception (49). Due to the lack of sufficient training data and the GPU memory constraint, we cannot apply 3D CNNs on complete CT volumes. In order to compare the proposed SLP-Net with other 3D deep learning architectures, we apply CT sequence data, which can be considered as 3D data, to train 3D models, including C3D (50), I3D (51), and S3D (52). We report the detection results for slice/sequence-level and case-level in **Table 2**. We applied a similar strategy to that in *Auxiliary Diagnosis With Attention Maps* section to determine the prediction result of a volume when using the 2D models. First, Grad-CAM was used to generate the activated maps of 2D slices, and binary activated maps can be obtained through thresholding. If five consecutive slices in a volume were predicted as indicative of viral pneumonia, and the intersection area of their activated area exceeds 50% of the union area, the volume was considered to be indicative of viral pneumonia.

**Table 2:** Classification results for VP, BP and normal controls by different methods.

Method	Slice/Sequence-Level					Case-level				
	Kappa	F1	B-Acc	Sen	Spe	Kappa	F1	B-Acc	Sen	Spe
AlexNet	0.5207	0.5680	0.6872	0.6375	0.7370	0.6889	0.7381	0.8207	0.8274	0.8140
VGG19	0.6258	0.6574	0.7502	0.7152	0.7853	0.7709	0.8000	0.8571	0.8690	0.8601
ResNet34	0.6767	0.7100	0.7948	0.7783	0.8112	0.8489	0.8598	0.9045	0.9048	0.9043
InceptionV3	0.5177	0.6107	0.7156	0.6978	0.7333	0.7692	0.7976	0.8607	0.8631	0.8582
Xception	0.6802	0.6776	0.7688	0.7252	0.8124	0.8500	0.8631	0.9048	0.9107	0.9061
C3D	0.7382	0.7442	0.8232	0.8013	0.8450	0.8616	0.8729	0.9158	0.9183	0.9132
I3D	0.7437	0.7513	0.8403	0.8302	0.8132	0.8481	0.8952	0.9229	0.9167	0.9290
S3D	0.7525	0.7332	0.8106	0.7696	0.8517	0.8696	0.8796	0.9297	0.9133	0.9157
**SLP-Net**	**0.8280**	**0.8123**	**0.8665**	**0.8397**	**0.8934**	**0.9263**	**0.9291**	**0.9523**	**0.9524**	**0.9521**

The best performance of all the methods is highlighted in bold.

**Figure 6:**
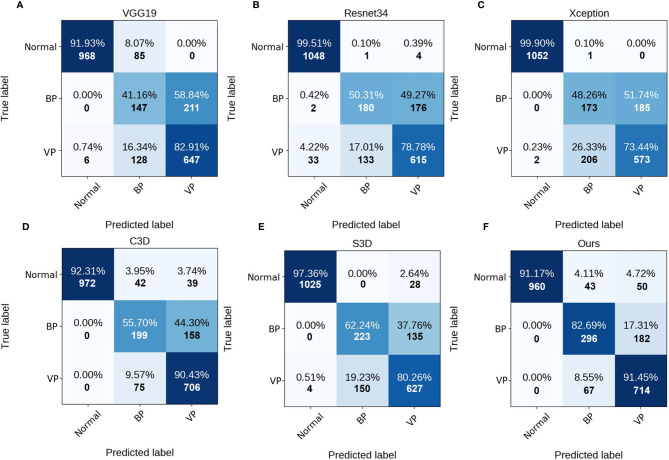
Confusion matrices of the different methods at slice/sequence level. **(A–F)** are the results of VGG19, Resnet34, Xception, C3D, S3D and ours, respectively. The numbers in the confusion matrices denote the percentage (above) and number (below) of the predicted class.

**Figure 7:**
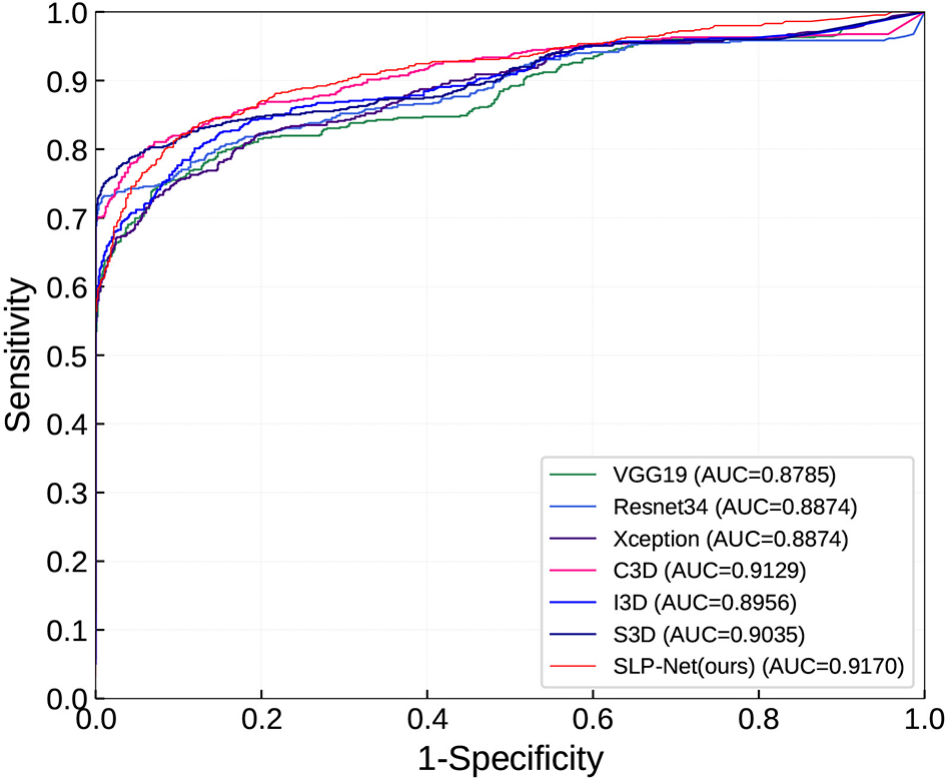
ROC curves in classifying viral pneumonia (VP) and bacterial pneumonia (BP) of the compared models and the proposed method.

As can be observed, the 3D networks achieve higher performances than the 2D networks, which confirms the importance of the combination of axial dimensional and spatial information for accurate detection results. At a slice/sequence level, our SLP-Net outperformed other methods in terms of *kappa*, *F*1 and *Sen* by a large margin, as well as achieving the best performance at a volume level. In addition, **Figure 6** shows the confusion matrices of VGG-19, Resnet34, Xception, C3D, S3D, and our method over the dataset. These results further indicate the superiority of the performance of our approach. As stated in the Introduction section, the difficulty of pneumonia diagnosis is the differentiation between BP and VP. Accordingly, we conducted experiments on the dataset contained VP and BP samples only. Results are shown in **Table 3** and **Figure 7**. We may observe that the proposed method again produces the best performance compared to the other methods. **Table 3** gathers all the performances of these models. The results show that the 3D-based method is generally better than the 2D-based method, mainly because the 3D input provides richer spatial information, which allows the model to learn and extract the subtle differences in the spatial distribution of different diseases, which is especially important for difficult samples with similar lesion appearance. In this regard, our proposed method not only utilizes the 3D information, but also explicitly focuses on the lesion area in the decision-making process through attention map, which makes the classification results more reliable. This idea can be applied to many different medical image-based classification tasks, since the similarity of lesion appearance is a problem in many scenarios.

**Table 3:** Comparison of different methods in classifying viral pneumonia (VP) and bacterial pneumonia (BP), at a slice/sequence level.

Method	Acc	Sen	Spe	AUC (*p*-value)
AlexNet	0.7327	0.8182	0.6650	0.8700 (*p* <0.001)
VGG19	0.7776	0.8197	0.7442	0.8785 (*p* <0.001)
ResNet34	0.7939	0.8305	0.7649	0.8874 (*p* <0.001)
InceptionV3	0.7429	0.8028	0.6955	0.8697 (*p* <0.001)
Xception	0.8170	0.7704	0.8438	0.8874 (*p* <0.001)
C3D	0.8218	**0.869**	0.7844	0.9129 (*p* <0.05)
I3D	0.8320	0.8274	0.8356	0.8956 (*p* <0.001)
S3D	0.8361	0.8413	0.8319	0.9035 (*p* <0.01)
**SLP-Net**	**0.8476**	0.8459	**0.8490**	**0.9170**

P-value is calculated by Delong’s test. The best performance of all the methods is highlighted in bold.

**Table 4** summarizes space and time cost of different methods. For fair comparison of inference time, we test all these models with PyTorch. Our SLP-Net had the best time efficiency and achieved smallest model size because it didn’t use 3D convolution operations.

**Table 4:** Model size and inference time of different methods.

	C3D	I3D	S3D	SLP-Net
Model size (MB)	39.2	48.7	42.3	34.4
Time (ms)	41.4	59.0	47.1	39.5

## 5 Analysis and Discussion

### 5.1 Sensitivities to Hyperparameters

In **Table 5** we investigate the sequence settings, i.e., *n* and *k*, denote the number of slices per sequence and the number of overlapping slices between two sequences, respectively. By default we set *n* = 10 and *k* = 5. As can be observed, the performance was greatly affected by the value assigned to *n*. When *n* is set very small (*n* = 5), the *kappa* and *F*1 drop by the considerable margin of 3%, demonstrating that the more axial dimensional information ConvLSTM encodes, the more the model benefits. However, when *n* is greater than 15, the model performance will decline. This may be due to the fact that as the slice number increases, there will be less training data, resulting in the model not being fully trained. **Table 5** also shows that our result is impacted just marginally when *k* is within a scale of 5-7. The performance of the model mainly depends on the abundance of the information contained in the sequence, that is, the more information contained in the sequence, the better the classification performance. Compared to n = 10, the sequence provide less timing information when n = 5, so the classification performance will decrease. If n is too large (e.g., n = 20), the performance will decrease due to fewer training samples.

**Table 5:** Effect of different settings of hyperparameter *n* and *k* on the results.

Method	kappa	F1	Acc	B-Acc	Sen	Spe
n = 5, k = 3	0.7376	0.7469	0.8546	0.8272	0.8096	0.8449
n = 10, k = 3	0.7652	0.7701	0.8684	0.8453	0.8307	0.8599
n = 10, k = 5	0.8241	0.8091	0.9012	0.8641	0.8471	0.8911
n = 10, k = 7	0.8280	0.8123	0.9034	0.8665	0.8397	0.8934
n = 15, k = 5	0.7453	0.7557	0.8577	0.8362	0.8231	0.8493
n = 20, k = 5	0.7329	0.7068	0.8577	0.7930	0.7450	0.8410

Here, *n* and *k* denote the number of slices in the sequence and the number of overlapping slices between two sequences, respectively.

### 5.2 Ablation Study

**Figure 8:**
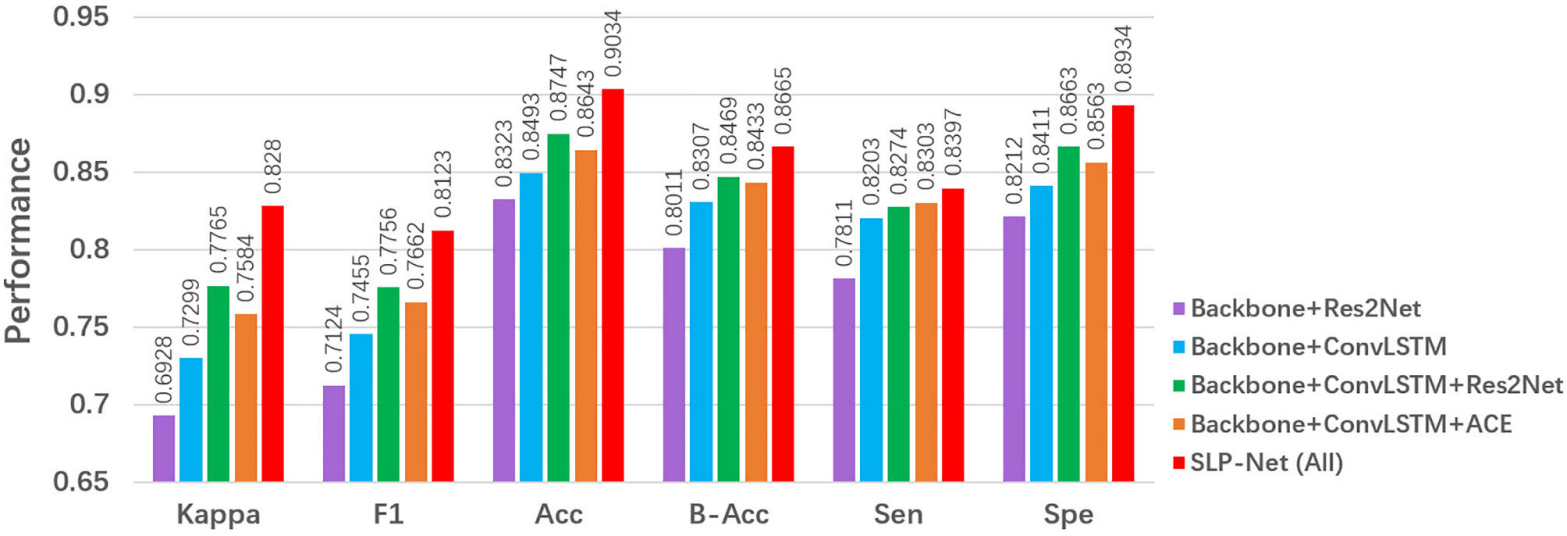
Ablation studies of our SLP-Net.

Our SLP-Net employs three main components to form the classification framework: a sequence CNNs with multi-scale receptive fields, a ConvLSTM module, and a carefully designed ACE loss. In this subsection, we analyze and discuss the network under different scenarios to validate the performance of each key component of our model, and the results of different combinations of these modules are reported in **Figure 8**.

#### 5.2.1 Effectiveness of ConvLSTM

To explore the contribution of the ConvLSTM, we use a ResNet50 pretrained on ImageNet as the backbone. As shown in **Figure 8**, a backbone + ConvLSTM + Res2Net method clearly outperformes the backbone + Res2Net, with improvement of about 6% in *F*1. This shows that the ConvLSTM is capable of extracting the axial dimensional and spatial information, thus memorizing the change in appearance that corresponds to axial dimensional information, and improving the performance in identifying and discriminating between VP and BP.

#### 5.2.2 Effectiveness of the Res2Net module

We investigated the importance of the multi-scale sequence module, i.e., Res2Net. From **Figure 8**, we observe that a backbone + ConvLSTM + Res2Net model outperformed the backbone model in terms of major metrics, i.e., *kappa* and *F*1. This suggests that introducing the Res2Net module enables the encoder to capture more discriminative features to accurately differentiate VP from BP.

#### 5.2.3 Effectiveness of ACE

Finally, we investigate the importance of the ACE loss. From the results in **Figure 8**, it may clearly be observed that the ACE Loss effectively improves the classification performance in our model. One possible reason is that, with the ACE loss, the ConvLSTM explores the axial dimensional dynamics of appearance features in CT sequences, and these features are further aggregated for classification purposes.

#### 5.2.4 Effectiveness of Attention Maps

To investigate the contribution of the Attention Map, we added an additional experiment—case-level classification without Attention Map. Specifically, sequence-level classification results were first obtained using SLP-Net, and if there were VP or BP sequences in a volume, the type with more number is used as the category of the whole volume. If it does not contain VP and BP sequence, it is classified as normal. Notably, if there are the same number of VP and BP sequences, the one with the greater average sequence probability value is treated as the dominant category. **Table 6** shows the result, where SLP-Net with Attention Map as auxiliary achieves better performance than without Attention Map. This demonstrates that with the aid of attention map, the distribution of lesions can be considered simultaneously in the decision-making process, thus improving the performance of case-level classification.

**Table 6:** Ablation study of Attention Map in classifying VP, BP, and Normal controls at the case-level.

Method	Kappa	F1	B-Acc	Sen	Spe
Without Attention Map	0.8731	0.9090	0.9167	0.9174	0.9160
With Attention Map	0.9263	0.9291	0.9523	0.9524	0.9521

## 6 Discussion and Conclusions

### 6.1 Limitations

Although our method achieves better results in the pneumonia classification task compared to other methods, this work still has some limitations. Firstly, we used the multiscale feature technique Res2Net in the feature extraction part, but did not further explore the hyperparameter settings in it, and although we believe that careful selection of hyperparameters may further improve the classification performance, no additional experiments were conducted in this work to compare the impact of different hyperparameters since this is not the focus of our work. Secondly, the model is not evaluated on an external dataset. To our knowledge, there are no publicly available 3D CT datasets for different types of pneumonia classification tasks, and it is difficult to collect compliant data from multiple centers due to various conditions. We intend to evaluated the performance of our model on external datasets in the future.

### 6.2 Conclusion

Hospitals are beginning to use CT imaging in the diagnosis of viral pneumonia, and it is vital to improve the sensitivity of diagnostic methods so as to reduce the incidence of false negatives. AI-empowered image acquisition workflows are effective, and may also aid in protecting clinicians from viral pneumonia (e.g., COVID-19) infection. Although several effective AI-based COVID-19 diagnosis or lesion segmentation methods have been introduced recently, automated differentiation of viral pneumonia from other types of pneumonia is still a challenging task. The motivation of this study was to employ AI techniques to alleviate the problem posed by the fact that even radiologists are hard pressed to distinguish VP from BP, as they share very similar presentations of infection lesion characteristics in CT images.

In this paper, we have proposed a novel viral pneumonia detection network, named SLP-Net. By contrast with previous 2D slice-based or 3D volume-based methods, we considered continuous CT images as time sequences. Our model first utilized the sequence CNNs with multi-scale receptive fields to extract a sequence of higher-level representations. The feature sequences were then fed into a ConvLSTM to capture axial dimensional features. Finally, in order to ensure that as many valid features from previous slice as possible are encoded into the later CT slices, a novel ACE loss was proposed to optimize the output of the SLP-Net. Furthermore, during the prediction phase, we used sequence attention maps for auxiliary classification to predict each volume, which can enhance the confidence level of the results. Overall, in order to accurately distinguish VP from BP and normal subjects, we used the sequence CNNs with multi-scale receptive fields to extract more differentiating features, and then applied a ConvLSTM to capture axial dimensional features of the CT sequence, thereby obtaining features containing both axial dimensional and spatial information. The superior evaluation performance achieved in the classification experiments demonstrate the ability of our model in the differential diagnosis of VP, BP and normal cases. Although we only evaluated our method on the CT dataset of pneumonia, it can be adapted to any other 3D medical image classification problems, such as lung cancer imaging analysis, and the identification of Alzheimer’s disease. In future work we will further validate our models on even larger datasets, and seek its implementation in real clinical settings.

## Data Availability Statement

The original contributions presented in the study are included in the article/supplementary material. Further inquiries can be directed to the corresponding author.

## Ethics Statement

The studies involving human participants were reviewed and approved by the Hwa Mei Hospital, University of Chinese Academy of Sciences. The patients/participants provided their written informed consent to participate in this study.

## Author Contributions

JH, JX, and RL were involved in data analysis and interpretation, and drafting and revising the manuscript. HH, YM, KY, RRL, YLZ, and JJZ were involved in data analysis and interpretation. JL, JFZ, and YTZ were involved in study conceptualization, supervision, revising the manuscript. All authors contributed to the article and approved the submitted version.

## Funding

This work was supported in part by the Zhejiang Provincial Natural Science Foundation of China (LZ19F010001, and LQ20F030002), in part by the Key Project of Ningbo Public Welfare Science and Technology (2021S107), and in part by the Youth Innovation Promotion Association CAS (2021298).

## Conflict of Interest

The authors declare that the research was conducted in the absence of any commercial or financial relationships that could be construed as a potential conflict of interest.

## Publisher’s Note

All claims expressed in this article are solely those of the authors and do not necessarily represent those of their affiliated organizations, or those of the publisher, the editors and the reviewers. Any product that may be evaluated in this article, or claim that may be made by its manufacturer, is not guaranteed or endorsed by the publisher.

## Footnotes

1
https://github.com/HzFu/COVID19.
